# Using growth velocity to predict child mortality^1^,^2^

**DOI:** 10.3945/ajcn.115.118679

**Published:** 2016-02-03

**Authors:** Catherine Schwinger, Lars T Fadnes, Jan Van den Broeck

**Affiliations:** 3 Center for International Health, Department of Global Public Health and Primary Care, and; 4 Department of Clinical Dentistry, University of Bergen, Bergen, Norway

**Keywords:** anthropometry, longitudinal growth, mortality, prediction, WHO growth velocity standards

## Abstract

**Background:** Growth assessment based on the WHO child growth velocity standards can potentially be used to predict adverse health outcomes. Nevertheless, there are very few studies on growth velocity to predict mortality.

**Objectives:** We aimed to determine the ability of various growth velocity measures to predict child death within 3 mo and to compare it with those of attained growth measures.

**Design:** Data from 5657 children <5 y old who were enrolled in a cohort study in the Democratic Republic of Congo were used. Children were measured up to 6 times in 3-mo intervals, and 246 (4.3%) children died during the study period. Generalized estimating equation (GEE) models informed the mortality risk within 3 mo for weight and length velocity *z* scores and 3-mo changes in midupper arm circumference (MUAC). We used receiver operating characteristic (ROC) curves to present balance in sensitivity and specificity to predict child death.

**Results:** GEE models showed that children had an exponential increase in the risk of dying with decreasing growth velocity in all 4 indexes (1.2- to 2.4-fold for every unit decrease). A length and weight velocity *z* score of <−3 was associated with an 11.8- and a 7.9-fold increase, respectively, in the RR of death in the subsequent 3-mo period (95% CIs: 3.9, 35.5, and 3.9, 16.2, respectively). Weight and length velocity *z* scores had better predictive abilities [area under the ROC curves (AUCs) of 0.67 and 0.69] than did weight-for-age (AUC: 0.57) and length-for-age (AUC: 0.52) *z* scores. Among wasted children (weight-for-height *z* score <−2), the AUC of weight velocity *z* scores was 0.87. Absolute MUAC performed best among the attained indexes (AUC: 0.63), but longitudinal assessment of MUAC-based indexes did not increase the predictive value.

**Conclusion:** Although repeated growth measures are slightly more complex to implement, their superiority in mortality-predictive abilities suggests that these could be used more for identifying children at increased risk of death.

See corresponding editorial on page 681.

## INTRODUCTION

Growth monitoring is nearly universally practiced in pediatric care worldwide (1) to detect growth faltering and thus intervene accordingly. Various anthropometric indexes, such as indexes based on weight, height, or midupper arm circumference (MUAC),^6^ have been suggested, but somewhat different abilities to predict the risk of child deaths were reported (2–8). Despite their hypothesized advantages for early detection of growth problems (9), and thus the possibility to direct life-saving interventions, currently only a few studies have assessed the mortality-predictive ability of longitudinal indicators of poor growth (i.e., growth velocities) (4, 10–12). Those studies used various approaches to define and score growth velocity. To our knowledge, only 1 study (4) looked at the predictive ability of weight velocity *z* scores (WVZs) by using the WHO standards. O'Neill et al. (4) found that very low weight velocity (*z* scores <−3) predicted death within 3 mo to some degree. However, they did not include WVZs in their comparative analysis and did not assess length velocity. In addition, changes in MUAC and MUAC *z* scores were not explored. This encouraged us to expand on the analysis of O'Neill et al. (4) within the same study population to further explore how various velocity measures could predict the risk of death.

We aimed to determine the ability of WVZs and length velocity *z* scores (LVZs) as well as changes in MUACZ and absolute MUAC (absMUAC) with the use of the WHO Child Growth Standards to predict child death within 3 mo in a cohort of children under the age of 5 y in the Democratic Republic of Congo (DRC). Second, we aimed to compare the mortality-predictive abilities of these indicators with those of attained growth.

## METHODS

**FIGURE 1:**
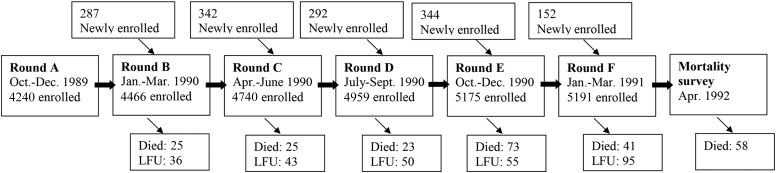
Study profile of the cohort study in Bwamanda, Democratic Republic of Congo: 1989–1991. LFU, lost to follow-up.

The primary study was a cohort study carried out in the health zone of Bwamanda in the northwest of the DRC from August 1989 to April 1991. The Bwamanda health zone is 1 of 16 administrative zones within the district of Sud-Ubangi, covering ∼10,000 km^2^. Details can be found elsewhere (13). In brief, 16 of a total of 52 villages in this health zone were randomly selected and all children <5 y living in the selected villages were enrolled. In 6 trimestral survey rounds a total of 5657 children were included (**Figure 1**).

Two trained field staff performed all anthropometric measurements according to a standardized protocol. Birth dates were available from road to health charts (a parent-held record of the child's identify, health, and development) or parent identity papers in 90% of the children. If these records were missing, the birth date was approximated after interviews with the mothers by using a local events calendar. Deaths were recorded in each survey round by interview of the caretakers and checked against death registries in the health centers and funeral registers kept by members of the clergy. After the sixth survey, in April 1991, a special mortality survey was conducted in April 1992 to record deaths up to that time.

### Ethics

The Bwamanda study was granted ethical approval from the Tropical Childcare Health Working Group at the University of Leuven (13). For this secondary analysis, ethical approval was obtained from the Ethical Committee at the University of Kinshasa (approval number: ESP/CE/008/14).

### Statistical analysis

Data were prepared with SPSS (version 19; IBM Corporation) and analyzed with Stata (version 13; StataCorp LP). We calculated *z* scores for weight-for-age (WAZ), height/length-for-age (LAZ), weight-for-height/length (WLZ), MUAC-for-age (MUACZ), weight velocity (WVZ), and length velocity (LVZ) using the WHO Child Growth Standards (9, 14, 15). To capture changes in absolute MUAC and MUACZs, the difference between 2 succeeding values was calculated, divided by the exact time period (in mo) between these values, and standardized to an exact 3-mo interval (multiplying the results by 3) [change in absMUAC (ΔabsMUAC), change in MUACZ (ΔMUACZ)]. During checks of data integrity, *z* scores of attained growth (WAZ, LAZ, WLZ, and MUACZ) exceeding ±6 *z* scores and *z* scores of longitudinal growth (WVZ, LVZ, and ΔMUACZ) exceeding ±10 *z* scores were considered implausible and set to missing. Higher cutoffs for weight and length velocity *z* scores were chosen, because the spread in growth velocity *z* scores usually is wider than in attained growth measures, even in healthy children (9). Values for ΔabsMUAC were set to missing if they were ±5 cm within 3 mo and were contrary to weight-based indexes. A total of 154 of 116,452 values (0.1%) for all indexes were discarded. We describe characteristics of nutritional status using median *z* scores (IQR). A Kaplan-Meier plot was constructed to present survival in this cohort. Reported *P* values are 2-sided, and *P* < 0.05 was considered significant.

**FIGURE 2:**
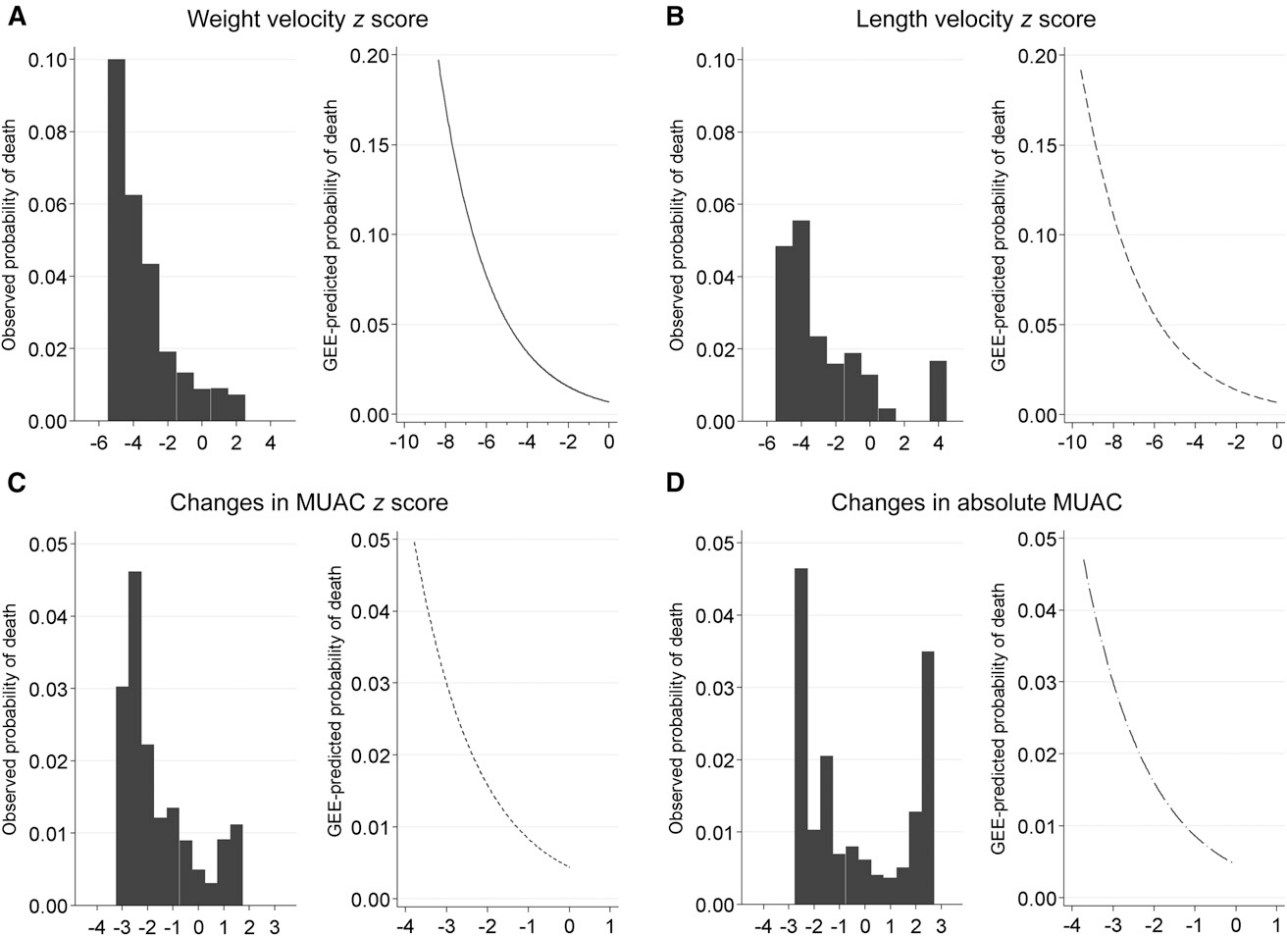
Observed and predicted (by using a GEE model) probabilities of death within 3 mo for children included in a cohort study in Bwamanda, Democratic Republic of Congo (1989–1991), for weight velocity *z* score (A; *n* = 2296), length velocity *z* score (B; *n* = 2296), change in MUAC-for-age *z* score (C; *n* = 4451), and changes in absolute MUAC (D; *n* = 4451). GEE, generalized estimating equation; MUAC, midupper arm circumference.

A generalized estimating equation model with a log link, a binomial distribution, an autoregressive correlation structure, and the unique child identification number as a cluster variable was constructed separately for each anthropometric index to assess the risk of death within 3 mo. In line with Briend et al. (16) that negative changes in growth could have negative health consequences even within the “normal range,” which was also indicated from a graphical assessment of the observed mortality risk in our data (**Figure 2**), we assessed the distance between expected values (*z* scores of 0) and observed anthropometric indexes in children with negative growth *z* scores. Thus, a continuous measure with the degree of negative scores was used in the continuous models. For the categorical models, we used the following categories for WVZ and LVZ: <−3 (severe), ≥−3 but <−2 (moderate), ≥−2 but <0 (mild), and ≥0 (reference). The Kasongo Project Team (17) found a higher risk of death if deceleration in MUAC was >1 SD. For comparative purposes, we chose the following categories for ΔMUACZ and ΔabsMUAC: <−1 (severe), ≥−1 but <−0.5 (moderate), ≥−0.5 but <0 (mild), and ≥0 (reference). Because a different effect of growth velocity on mortality would be expected when a child is well nourished compared with when he or she is malnourished already, we additionally report RRs adjusted for nutritional status at the beginning of the assessment period. In addition, the risk according to the age groups 0–6, 7–12, and 13–24 mo was assessed. Receiver operating characteristic (ROC) curves were used to present balance in sensitivity and specificity to predict child death between different thresholds of various growth measures. Analyses were restricted to age ranges available in the WHO standards (WVZ and LVZ: 0–24 mo; ΔMUACZ: 3–60 mo). It is recommended to interpret growth velocity in combination with attained growth. Therefore, we also calculated AUC values according to attained growth (stunted: LAZ <−2; wasted: WLZ <−2) at the beginning of the increment period. For the comparative analysis, we restricted analysis to the age range that is common for all indexes (3–24 mo). AUCs of the different prediction models with the individual child as a cluster variable were contrasted with the “roccomp” function in Stata. Sensitivity (i.e., percentage of children below the threshold among those who are dying), specificity (i.e., percentage of children above the threshold among those who are not dying), positive-predictive value (i.e., percentage of children who are dying among those who are below the threshold), and negative-predictive value (i.e., percentage of children who are not dying among those who are above the threshold) for certain cutoffs are presented. Optimal cutoffs for velocity indexes are defined as the value with the largest product of sensitivity and specificity.

## RESULTS

### Description of the sample

The study sample comprised 5657 children, of whom 51.3% were male and 98% were from the ethnic group Ngbaka. They were, on average, 36 mo old (SD: 21 mo). Children were measured up to 6 times with, on average, 3 mo between measurements. The median follow-up time was 15 mo. This provided 5294 WVZs, 5086 LVZs, 14,065 changes in MUACZs (ΔMUACZ), and 17,851 changes in absMUAC (ΔabsMUAC) for analysis. The nutritional status of the study sample is summarized in **Table 1**.

**TABLE 1:** Mean nutritional status according to age category of 5657 children <5 y included in a cohort study in Bwamanda, Democratic Republic of Congo: 1989–1991^1^

	0–6 mo		7–12 mo		13–24 mo	
	Mean ± SD	*n*	Mean ± SD	*n*	Mean ± SD	*n*
WAZ	−0.8 ± 1.2	1977	−1.5 ± 1.2	2015	−1.5 ± 1.1	4405
LAZ	−1.2 ± 1.4	1951	−1.9 ± 1.3	1985	−2.4 ± 1.2	4361
WLZ	0.2 ± 1.4	1945	−0.4 ± 1.2	1983	−0.4 ± 1.0	4349
MUACZ	−1.5 ± 1.1	1219	−1.9 ± 1.1	2015	−2.0 ± 1.1	4416
absMUAC	11.6 ± 1.3	1980	12.3 ± 1.1	2015	12.6 ± 1.1	4416
WVZ	−1.0 ± 1.6	700	−1.1 ± 1.5	1501	−0.4 ± 1.5	3092
LVZ	−1.1 ± 2.1	679	−1.0 ± 2.0	1454	−0.8 ± 1.8	2952
ΔMUACZ	−0.4 ± 1.1	157	−0.2 ± 0.8	1472	−0.1 ± 0.8	3246
ΔabsMUAC	1.1 ± 1.4	704	0.1 ± 0.8	1503	−0.01 ± 0.8	3246

1 absMUAC, absolute midupper arm circumference; LAZ, height/length-for-age *z* score; LVZ, length velocity *z* score; MUACZ, midupper arm circumference-for-age *z* score; WAZ, weight-for-age *z* score; WLZ, weight-for-height/length *z* score; WVZ, weight velocity *z* score; ΔabsMUAC, change in absolute midupper arm circumference; ΔMUACZ, change in midupper arm circumference-for-age *z* score.

**FIGURE 3:**
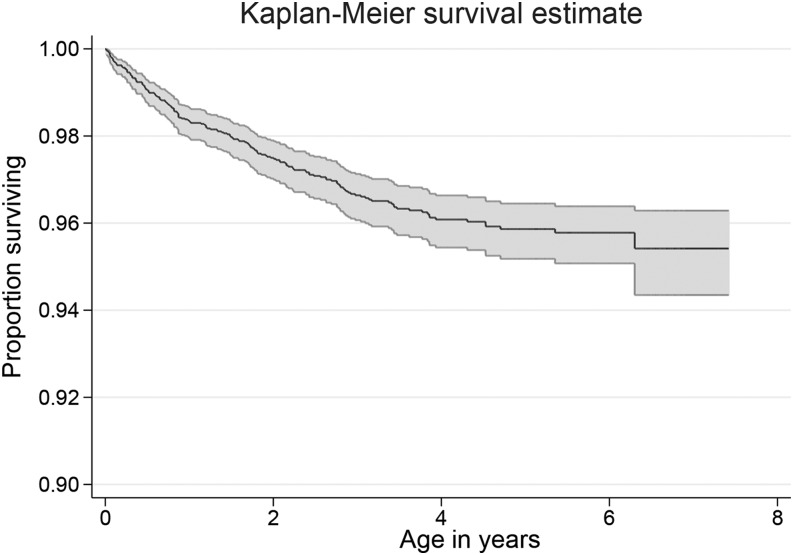
Kaplan-Meier survival curve showing survival for all 5657 children under the age of 5 y included in a cohort study in Bwamanda, Democratic Republic of Congo (1989–1991), at different ages (with 95% CIs).

Of all of the children, 246 (4.3%) died, 56% of whom were male. For 40 (16%) of these children anthropometric data in the 3-mo period before death were not available. The median age of death was 12 mo (IQR: 5.3, 26.1 mo; **Figure 3**). Causes of death are described elsewhere (18).

### Mortality risk by level of anthropometric index

For each anthropometric index, a separate generalized estimating equation regression model was constructed. **Table 2** summarizes the results for all velocity indexes either as continuous or as categorical variables. Independent of the attained growth status at the beginning of the increment period, there was a 1.5-fold increase in the risk of dying for weight velocity, a 1.2-fold increase for length velocity, and a 2.3-fold increase for ΔMUACZ for every *z* score falling under the *z* score of 0. For every centimeter negative change in absMUAC within 3 mo, the risk of dying increased by 2.4. An LVZ and a WVZ of less than −3 were associated with 11.8- and 7.9-fold increases, respectively, in the RR of death in the subsequent 3-mo period (95% CIs: 3.9, 35.5, and 3.9, 16.2, respectively). For velocity measures there was no trend in risk of dying according to age category. Estimates according to age category and for attained growth indexes can be found in the **Supplemental Tables 1** and** 2**). Figure 2 shows the observed and predicted risk curves for all velocity indexes, and **Supplemental Figure 1** shows the predicted risk curves for all attained and velocity indexes.

**TABLE 2:** RRs (95% CIs) for mortality for continuous and categorical growth velocity *z* scores of 5657 children <5 y of age included in a cohort study in Bwamanda, Democratic Republic of Congo (1989–1991), after GEE-based regression^1^

Index and category	*n*	RR (95% CI)^2^	Adjusted RR (95% CI)^3^
Continuous			
WVZ	—	1.49 (1.37, 1.62)	1.49 (1.30, 1.71)
LVZ	—	1.41 (1.30, 1.53)	1.24 (1.06, 1.45)
ΔMUACZ	—	1.90 (1.60, 2.26)	2.28 (1.74, 2.99)
ΔabsMUAC	—	1.87 (1.51, 2.31)	2.40 (1.84, 3.14)
WVZ			
Reference	1736	1	1
Mild	2688	1.34 (0.72, 2.51)	1.39 (0.73, 2.66)
Moderate	521	2.36 (1.06, 5.25)	2.65 (1.17, 6.04)
Severe	349	6.70 (3.42, 13.12)	7.92 (3.87, 16.19)
LVZ			
Reference	2741	1	1
Mild	1106	5.81 (2.14, 15.73)	5.82 (2.13, 15.94)
Moderate	622	5.82 (1.89, 17.97)	5.86 (1.86, 18.48)
Severe	603	11.70 (4.13, 33.17)	11.80 (3.92, 35.54)
ΔMUACZ			
Reference	6199	1	1
Mild	4029	1.42 (0.82, 2.46)	1.80 (1.03, 3.15)
Moderate	2347	2.12 (1.20, 3.75)	3.10 (1.69, 5.68)
Severe	1486	3.66 (2.10, 6.38)	6.25 (3.26, 11.98)
ΔabsMUAC			
Reference	8718	1	1
Mild	4214	1.68 (1.01, 2.79)	2.59 (1.54, 4.37)
Moderate	2504	1.97 (1.13, 3.43)	3.80 (2.08, 6.98)
Severe	1325	2.74 (1.50, 5.01)	6.13 (3.18, 11.82)

1 *z* Score categories for WVZ and LVZ are defined as follows: reference group, ≥0; mild, <0 but ≥−2; moderate, <−2 but ≥−3; and severe, <−3. For the MUAC-based indexes, these are as follows: reference, ≥0; mild, <0 but ≥−0.5; moderate, <−0.5 but ≥−1; and severe, <−1. GEE, generalized estimating equation; LAZ, length-for-age *z* score; LVZ, length velocity *z* score; MUAC, midupper arm circumference; WLZ, weight-for-length *z* score; WVZ, weight velocity *z* score; ΔabsMUAC, change in absolute midupper arm circumference; ΔMUACZ, change in midupper arm circumference-for-age *z* score.

2 For simplicity, continuous variables are converted so that 1 unit is a decrease in 1 *z* score. The RR represents the risk of death with each *z* score falling under the *z* score of 0. For absolute MUAC, the RR represents the risk of death for every centimeter decrease compared with an MUAC of ≥12.5 cm.

3 Adjusted for attained growth (WLZ, LAZ, or MUAC, respectively) at the beginning of the increment period.

### Comparison of abilities to predict death

**FIGURE 4:**
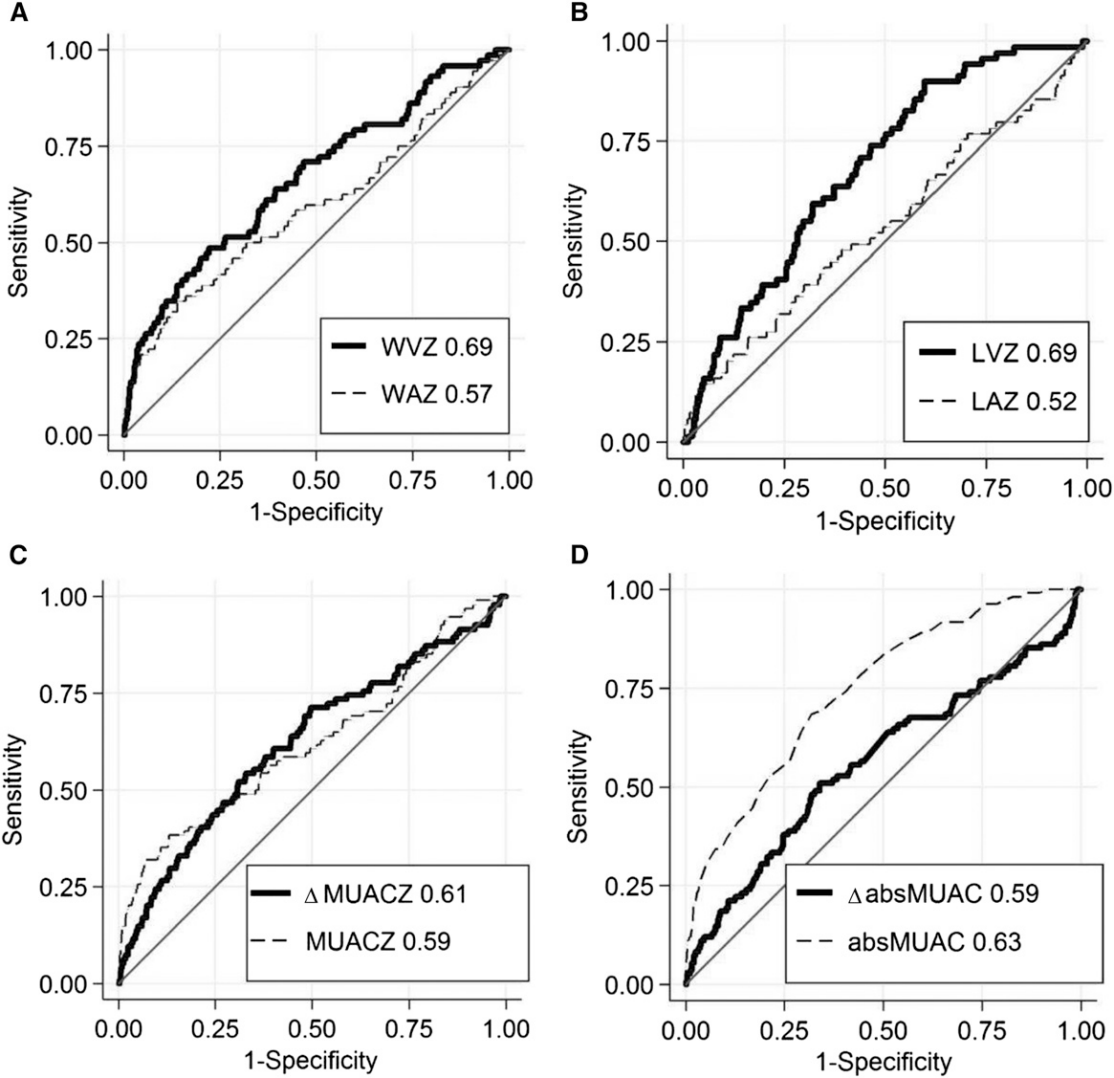
Receiver operating characteristic curves for ability to predict death within 3 mo in 2567 children aged 3–24 mo included in a cohort study in Bwamanda, Democratic Republic of Congo (1989–1991), by WVZ and WAZ (A), LVZ and LAZ (B), ΔMUACZ and MUACZ (C), and ΔabsMUAC and absMUAC (D). *z* Scores are calculated with help of the WHO Child Growth Standard. AUC values of the individual predictors are given in the inserts of each plot. absMUAC, absolute midupper arm circumference; LAZ, length-for-age *z* score; LVZ, length velocity *z* score; MUAC, midupper arm circumference; MUACZ, midupper arm circumference *z* score; WAZ, weight-for-age *z* score; WVZ, weight velocity *z* score; ΔabsMUAC, change in absolute values of midupper arm circumference; ΔMUACZ, change in midupper arm circumference *z* score.

Balances between sensitivity and specificity to predict child death at different thresholds are presented with ROC curves in **Figure 4**. Length and weight velocities showed the largest AUCs, with 0.69 and 0.67, respectively. The corresponding values for ΔMUACZ (0.61) and ΔabsMUAC (0.59) were slightly lower (*P* < 0.05). A comparative analysis showed that velocity *z* scores for weight and length performed significantly better in predicting deaths than did their attained counterparts (WAZ: 0.57; *P* < 0.05; LAZ: 0.52; *P* < 0.001). For absMUAC, the attained index was superior to the changes in absMUAC (*P* < 0.001), and for MUACZ no difference was shown (*P* = 0.97) (see Figure 4). AUC values did not differ between those who were ≤6 mo and those who were 7–24 mo old. Restricting analysis to the group of children who were stunted (LAZ <−2) at the beginning of the assessment period, the AUC for length velocity increased to 0.74. In the group of wasted children (WLZ <−2), the AUC for weight velocity increased to 0.87 and for absMUAC to 0.71. Exact AUC values with 95% CIs can be found in **Supplemental Table 3**. Sensitivity, specificity, and positive- and negative-predictive values extended the ROC curve analysis and are presented in **Supplemental Table 4**.

## DISCUSSION

This article showed that growth velocity measures can be useful in identifying a high risk of dying among children <5 y old living in the DRC. There was an exponential increase in the risk of dying with decreasing growth velocity in all 4 indexes. Comparing the mortality-predictive abilities of the 4 velocity indexes with the help of ROC curves and contrasting them to those of attained indexes, LVZ, WVZ, and absMUAC performed best. The predictive ability could be improved if attained growth at the beginning of the increment period was taken into account.

We are aware of only one other publication on the mortality-predictive ability of growth velocity that used WHO Child Growth Standards (4). Although the authors used the same study population as we did, their objective was restricted to weight velocity and noncomparative analyses only. Nevertheless, it is important to compare the velocity indexes to the more traditionally used indexes of attained growth (WAZ, LAZ, and WLZ) to provide a basis for a discussion about their usefulness. Two other studies that investigated the relation between mortality and weight velocity found a superiority of attained indexes (10, 11). Neither of the studies scored velocity according to an external reference and both were carried out within the framework of treatment programs, which could have biased the growth velocities, especially of those children with the worst nutritional status at baseline.

With growth velocities there is usually a trade-off concerning the length of the assessment period. The shorter the period, the higher the (physiologic) variability in growth velocity and the higher the measurement error compared with the actual growth. But the longer a growth velocity period extends, the less relevant it is for the current situation and the more it approaches attained growth, which is more of a cumulative growth measure. This is exemplified and discussed by Bairagi et al. (10), who looked at 1-y growth increments and found an inferiority of weight and height velocities compared with weight-for-age, height-for-age, and weight-for-height. However, they also found that weight velocity was reduced just before death and stated that velocities might be a good discriminator of short-term mortality. This was also emphasized by the Kasongo Project Team (17), where growth deceleration over 2–4 mo increased the mortality risk within the subsequent 100 d, whereas an association between death and attained indexes could not be shown with the same study population (19). Although single velocity measures are often critiqued because of their high variability in consecutive periods, in our analysis the discriminating power of 3-mo velocity *z* scores for weight and length to predict short-term death was better than that of conventionally used approaches.

It could be argued that growth velocity is more complex than attained growth measures, which could be a challenge for the applicability in the field. For the calculation, at least 2 measurements are needed. This requires more effort and might not be possible in some situations [e.g., in acute (clinical) settings]. Still, even with attained growth measures, assessing a trend of the individual’s growth measures rather than 1 separate value alone is more valuable for different reasons (20). A low value at 1 time point cannot discriminate those who are small by constitution from those whose growth curve has decreased recently. In addition, a single low measurement indicates that the child is already obviously malnourished and therefore treatment would be unnecessarily delayed (21). The first signs of growth faltering are growth curves that cross a *z* score line that are decreasing sharply or are flattening (22). However, a visual assessment of growth faltering can be more difficult to perform objectively, especially for those with less experience. This again leads to a disproportionate focus on attained values rather than the development over time (23). Growth velocity scores, however, could be an objective measure to quantify these trends.

Another disadvantage of growth velocity is that, currently, mostly statistical packages have been used to calculate velocity scores. There is also a scarcity of programs and tools for instant plotting of scores. Nevertheless, with rapid advances in the field of mobile technology and the spread of smart-phone use, it should be feasible to create an easy-to-use application to calculate and visualize growth velocity *z* scores, which could guide clinical decision making.

There are methodologic limitations that need to be mentioned. One relates to our definition of velocity in MUAC. No velocity standards for MUAC are available in the currently recommended set of WHO standards and therefore we standardized changes to a 3-mo period. This does not account for the different growth rates at different ages and could dilute our estimates. The absMUAC had a higher AUC than changes in absMUAC or MUACZs in our study. In addition, other studies have not found any advantages of scoring MUAC according to age (24, 25), assuming that a higher selection of younger children, who are also more vulnerable to deaths partly unrelated to nutritional status, adds to the mortality-predictive ability (3, 4). The mechanisms that put younger children at higher risk of dying unrelated to nutritional status might also be one of the reasons for the relatively low AUC values as well as low sensitivity and specificity in our study. However, with a threshold in LVZ of <−1, the sensitivity of death as an outcome was 71% and the specificity 54%. We are not aware of other feasible tests applicable to the population level in this setting that are known to have better predictive effects. Due to the availability of the WHO growth standards we restricted analysis to ages 3–24 mo, thus assessing a relatively young study sample. In a hospital-based study in Niger, the prognostic accuracy of weight-for-height was better in older children (34–59 mo) (6). Within our sample, there was no difference in predictive ability between those aged <6 mo and those aged 7–24 mo. Previous analysis showed that for 13% of the deaths no cause could be cited (13). Those could have been from factors unrelated to nutritional status (e.g., accidents) and thereby dilute our estimates. The main causes of death in our sample were malaria and acute respiratory infections (43%). Even though malnutrition adds vulnerability to these children, these illnesses are not as strongly related to nutrition as, for example, diarrhea and might therefore slightly reduce the AUC, sensitivity, and specificity values in our analysis. To further generalize the findings, predictive abilities of the velocity scores need to be tested in other settings with a different disease pattern. The positive-predictive value is dependent on the prevalence of the outcome. Mortality, although relatively high in our study sample, is still a rare outcome and low positive-predictive values are expected.

The aim of this assessment was to predict the risk of child death on the basis of anthropometric measurements and not to establish a net of causal relations. Therefore, we did not adjust our analyses for other factors, such as socioeconomic status or morbidity. The data used are relatively old and the risk of mortality could have changed slightly since then. However, there is a lack of recent studies available that could enable a similar assessment, related to the required methodologic rigor of the anthropometric measurements, sufficient frequency, and length of follow-up combined with a study size of required power to enable assessment of mortality. This study adds knowledge about children who are particularly vulnerable and important to reach with appropriate interventions to reduce child mortality.

In summary, our study provides evidence that growth velocity *z* scores according to WHO standards could be useful and important as a tool in identifying children at high risk of negative health consequences such as death. Growth velocity *z* scores were better able to predict death in the short term (i.e., within 3 mo) than the more conventionally used attained growth counterparts. The combination of growth velocity and attained growth produced the best results. This suggests that WVZs and LVZs could be used more to identify children at risk of dying. However, growth velocity assessment requires repeated measures and is thus slightly more complex to implement than the use of attained growth measures. Repeated anthropometric measurements might be difficult in some settings in which a lack of resources, such as material and competent and motivated staff, or a lack of infrastructure restrict effective actions.

## Footnotes

6 Abbreviations used: absMUAC, absolute midupper arm circumference; DRC, Democratic Republic of Congo; LAZ, length-for-age *z* score (also applied for height-for-age); LVZ, length velocity *z* score; MUAC, midupper arm circumference; MUACZ, midupper arm circumference *z* score; ROC, receiver operating characteristic; WAZ, weight-for-age *z* score; WLZ, weight-for-length *z* score (also applied for weight-for-height); WVZ, weight velocity *z* score; Δ, change.
